# Pre-travel vaccine information needs, attitudes, drivers of uptake and the role for decision aids in travel medicine

**DOI:** 10.1093/jtm/taad056

**Published:** 2023-04-19

**Authors:** Sarah L McGuinness, Owen Eades, Holly Seale, Allen C Cheng, Karin Leder

**Affiliations:** School of Public Health and Preventive Medicine, Monash University, Melbourne 3004, Australia; Department of Infectious Diseases, The Alfred Hospital and Central Clinical School, Monash University, Melbourne 3004, Australia; School of Public Health and Preventive Medicine, Monash University, Melbourne 3004, Australia; Department of Infectious Diseases, The Alfred Hospital and Central Clinical School, Monash University, Melbourne 3004, Australia; School of Population Health, Faculty of Medicine and Health, University of New South Wales, Sydney 2052, Australia; School of Public Health and Preventive Medicine, Monash University, Melbourne 3004, Australia; Monash Infectious Diseases Service, Monash Health and School of Clinical Sciences, Monash University, Melbourne 3168, Australia; School of Public Health and Preventive Medicine, Monash University, Melbourne 3004, Australia; Victorian Infectious Diseases Service, Royal Melbourne Hospital at the Peter Doherty Institute for Infection and Immunity, Melbourne 3000, Australia

**Keywords:** vaccines, Pretravel, travellers, decision-making, vaccine confidence, trust, health information

## Abstract

**Background:**

Many travellers do not receive vaccines pre-travel. Tools such as vaccine decision aids could support informed vaccine decision-making. We aimed to characterise Australians’ pre-travel vaccine attitudes, behaviours and information needs and examine the role for decision aids in travel medicine.

**Methods:**

Online cross-sectional survey of Australian adults in December 2022. We included questions on demographics, pre-travel health-seeking behaviour, and information needs. We measured vaccine confidence (Vaccine Confidence Index Index) and used hypothetical disease scenarios to evaluate behavioural and social drivers of vaccination. We used multivariable logistic regression models to identify predictors of vaccine uptake and thematically analysed free-text responses.

**Results:**

We received complete survey responses from 1223/1326 Australians (92% response rate). Amongst those reporting previous overseas travel, 67% (778/1161) reported past pre-travel health encounter(s) and 64% (743/1161) reported past pre-travel vaccination. Half (50%) strongly agreed that vaccines were important for their health; fewer strongly agreed that vaccines were safe (37%) and effective (38%). In multivariable analyses, past pre-travel vaccine uptake was associated with increasing age (OR = 1.17 [95% CI 1.08–1.27] p < 0.001 per ten-year increase) and travel to higher-risk destinations (OR = 2.92 [2.17–3.93] p < 0.001); travellers visiting friends and relatives (VFRs) were less likely to have received pre-travel vaccines (OR = 0.74 [0.56–0.97] p = 0.028). Predictors for wanting vaccination against hypothetical diseases included past pre-travel vaccination (Disease X: OR 2.60 [1.91–3.56] p < 0.001) and confidence in vaccine safety (Disease X: OR 7.18 [5.07–10.18], p < 0.001); past VFR travel was predictive of not wanting vaccination (Disease X: OR 0.72 [0.52–1.00], p = 0.049). Most (63%) were interested in using a vaccine decision aid, generally together with a trusted health professional.

**Conclusions:**

Health professionals play an important role in supporting pre-travel vaccine decision-making. However, our findings indicate that reliable, accurate and engaging digital resources, such as decision aids, could support travellers to make informed pre-travel vaccine decisions.

## Introduction

Travellers are potentially at higher risk of a broad range of infectious diseases and play a key role in their global spread.^1^ Some travel-related infections can be prevented through vaccination, but many travellers fail to seek or receive vaccines pre-travel. While factors thought to contribute to poor pre-travel vaccine uptake include low disease risk perceptions and vaccination costs (both in terms of willingness and capacity to pay),^2–4^ the determinants of individual travellers’ decisions around pre-travel vaccination are largely unknown.

The WHO Behavioural and Social Drivers (BeSD) of vaccination model provides a framework for understanding factors that motivate people to get vaccinated, and the practical or logistical hurdles to subsequent vaccine uptake.^5^ The BeSD has been used to understand barriers and enablers to uptake of childhood and COVID-19 vaccines, but to our knowledge has not previously been applied in a travel medicine context.

The COVID-19 pandemic has drawn attention to vaccine-preventable diseases (VPDs) and highlighted increasingly polarised vaccine attitudes, emphasising the need for effective health communication tools that can counter vaccine misinformation and support vaccine-related decision-making.^6^ Given the increasing role of the internet in health-related decision-making,^7^ there are potential advantages to utilising tools that can be delivered in a digital format, such as decision aids.^8^ Decision aids are evidence-based tools designed to offer patients reliable information on vaccine risks and benefits, help them clarify and communicate personal values, and guide them through decision-making processes; they can also facilitate shared-decision making with healthcare providers.^9^ Decision aids for influenza and MMR vaccines have been shown to reduce decisional conflict and increase vaccine uptake, but no studies have reported on development or evaluation of decision aids for travel-related VPDs.^10^

We aimed to characterise the attitudes, behaviours and information needs of a nationally representative cross-sectional sample of Australians towards vaccines generally and travel vaccines specifically.

## Methods

### Study design

This study, part of the broader TRAvel VAccine Decision Aids for Decision-making (TRAVAID) Project, aimed to investigate Australians’ pre-travel information- and health-seeking behaviours regarding pre-travel vaccines, drivers of past travel vaccine uptake, and information needs and attitudes relating to vaccine decision aids. Study reporting conforms to the STROBE Checklist for cross-sectional studies.^11^

We conducted an online survey of Australian adults in December 2022 with participants recruited through Dynata (https://www.dynata.com/), a health market research company. Dynata’s consumer research panel includes > 400 000 Australians recruited through thousands of websites, social media platforms, mobile applications and brand loyalty programs to obtain a diverse and representative sample. Panel members undergo a verification process to ensure survey responses are reliable, accurate and unique and data obtained undergoes regular quality checks, with removal of panellists providing illogical responses or spending insufficient time on surveys. Panel members completing surveys receive points that can be exchanged for rewards such as gift cards.

We collected and managed survey data online through REDCap,^12^ a secure web-based platform. Dynata distributed our survey link to a random sample of panel members residing in Australia. Panel members who opened the link and provided informed consent were screened for eligibility, with criteria comprised of age 18 years or older, Australian residence, speaking English and a history of overseas travel or plans to travel overseas within 2 years. If a participant began but did not complete the survey, we considered their consent withdrawn and did not use their data. Survey completion took between 8 and 10 minutes.

### Survey questions

We used closed- and open-ended questions to gather data on past travel experiences and demographics including age, gender, country of birth and jurisdiction of residence (see Supplementary Information). Our primary outcome, past uptake of pre-travel vaccination, was assessed using the question ‘Have you ever received vaccines before an overseas trip?’. We included questions from the vaccine literature, including three Vaccine Confidence Index (VCI) questions measuring general perceptions of vaccine importance, safety and efficacy.^13^ Participants were asked to rate their agreement (using a 5-point Likert scale from strongly agree to strongly disagree) with the following statements: ‘overall, I think vaccines are important for my health’, ‘overall, I think vaccines are safe’, and ‘overall, I think vaccines are effective’. We also adapted questions from another study^14^ to assess trust in vaccine information from different sources.

Questions from the WHO Behavioural and Social Drivers of vaccine uptake (BeSD) framework were adapted and included as part of two hypothetical scenarios (Table S1).^15^ Scenario 1 described ‘Disease X’ which had similar characteristics to influenza; a common but mild disease with a corresponding vaccine that was moderately efficacious, safe, low-cost ($20AUD) and would provide protection for around 6 months. Scenario 2 described as ‘Disease Y’ which had similar characteristics to yellow fever; a rare but high consequence disease with a corresponding vaccine that was highly efficacious, expensive ($200AUD), provided lifelong protection and was generally safe but with a 1 in 1 000 000 risk of death. For each scenario, respondents were asked to envision a 2-week trip to a risk area and indicate a) if they would want to be vaccinated pre-travel, b) if their close family and friends would want them to be vaccinated and c) how easy it would be to pay for the vaccine. The final section of the survey inquired about vaccine decision-making processes and respondents’ information needs and preferences regarding a vaccine decision aid. Two open-ended questions were posed: ‘What additional information would you find most useful in a vaccine decision aid?’, and ‘Is there anything else you would like to tell us?’

### Analysis

Data were entered into a REDCap database^12^ on a secure Monash University server and analysed using Stata (version 14). Implausible responses (e.g. age = 404) were classed as missing but the remainder of participants’ responses were retained. Descriptive and inferential analyses were performed. We used Pearson Chi-Square tests for univariate analyses and multivariable logistic models to identify i) predictors of past vaccination uptake and ii) wanting to be vaccinated against hypothetical diseases, with results presented as odds ratios with 95% confidence intervals and p-values. In multivariable analyses, predictor variables were dichotomised, with the exception of age (divided into ten-year age groups) and past number of trips (four categories: 1–2, 3–4, 5–10 and > 10 trips). Past travel destinations were dichotomised into higher-risk regions (Asia, Africa, Central America, South America, or the Middle East) and lower-risk regions (North America, Europe, Oceania, or Antarctica). Free-text responses to the two open-ended questions were managed in Excel and analysed inductively by two researchers (SLM and OE) using thematic analysis.^16^

### Sample size

We powered the study to allow analysis with a 95% confidence interval around the point estimate, allowing for a 3% margin of error; this required a sample size of at least 1067 people.^17^^,^^18^

## Results

**Figure 1:**
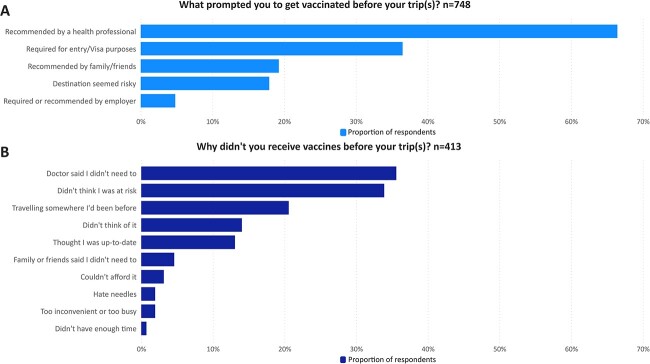
Reported reasons for receiving (panel A) or not receiving (panel B) vaccines pre-travel. Note: more than one response was allowed.

Of 1591 Australian adults assessed for eligibility, 1326 met eligibility criteria and 1223 (92%) completed the survey and were included in analyses (Figure S1). Median age was 64 (range 18–92), just over half were female (n = 628; 51%) and one-third were born overseas (n = 408; 33%) (Table 1). Jurisdiction-based representation was reflective of the Australian population. There was minimal missing data. Most (n = 1161; 95%) reported a history of overseas travel, frequently with 5 or more previous trips (n = 743; 64%) and overseas travel within the last 5 years (n = 720; 62%). Amongst those reporting overseas travel, 67% (n = 778) reported one or more pre-travel health encounters and 64% (n = 748) reported receiving at least one pre-travel vaccine; most pre-travel vaccines (n = 585; 78%) were received at respondents’ regular General Practitioner (GP) clinic. Positive and negative recommendations from a health professional were influential in patients receiving or not receiving pre-travel vaccination (Figure 1).

**Table 1:** Demographic and travel characteristics of 1223 study participants

	n (%)
**DEMOGRAPHIC CHARACTERISTICS** ^*^	
Gender	
Female	628 (51.4)
Male	592 (48.4)
Other^a^	3 (0.2)
Mean age, years (SD)	59.38 (16.54)
Median age, years (range; IQR)	64 (18–92)(46–72)
Age group (years)	
18–44	278 (22.8)
45–64	346 (28.3)
65 and older	598 (48.9)
Country of birth	
Australian-born	813 (66.6)
Overseas-born	408 (33.4)
Parent’s country of birth	
Australian-born	617 (50.5)
One or more parents overseas-born	606 (49.5)
Language other than English spoken at home	
Yes	182 (14.9)
No (English only)	1041 (85.1)
Jurisdiction of residence	
Australian Capital Territory (ACT)	26 (2.1)
New South Wales (NSW)	368 (30.1)
Northern Territory (NT)	9 (0.7)
Queensland (QLD)	272 (22.2)
South Australia (SA)	102 (8.3)
Tasmania (TAS)	28 (2.3)
Victoria (VIC)	318 (26.0)
Western Australia (WA)	100 (8.2)
Employment status	
Full-time	318 (26.0)
Part-time	146 (11.9)
Casual	49 (4.0)
Retired	564 (46.1)
Unemployed	42 (3.4)
Other^b^	104 (8.5)
Private health insurance	
Yes	719 (58.8)
No	503 (41.2)
TRAVEL CHARACTERISTICS	
Travel experience	
Number of overseas trips: 0	62 (5.1)
Number of overseas trips: 1–2	224 (19.3)
Number of overseas trips: 3–4	194 (16.7)
Number of overseas trips: > 5	743 (64.0)
Year of most recent trip for those with past overseas travel (n = 1161)	
Within last 5 years (since 2018)	720 (62.0)
More than 5 years age (prior to 2018)	441 (38.0)
Planning to travel overseas in next 2 years	
Yes	798 (65.3)
No	425 (34.7)
Regions visited (n = **1161)**^**c**^	
Asia	728 (62.7)
South Asia	197 (16.9)
Africa	167 (14.4)
North America	494 (42.5)
Central/South America	167 (14.4)
Europe	737 (63.5)
Middle East	205 (17.7)
Oceania^e^	767 (66.0)
Antarctica	26 (2.2)
Main purpose of travel, last trip (n = 1161)	
Tourism	727 (62.6)
Visiting friends and relatives (VFR)	358 (30.8)
Business	48 (4.1)
Other	28 (2.4)
Received pre-travel advice from a health professional (n = 1161)	
Yes	778 (67.0)
No	383 (33.0)
Received pre-travel vaccines (n = 1161)	
Yes	748 (64.4)
No	413 (35.6)
Location of pre-travel vaccination (n = 748)	
Regular GP clinic	585 (78.2)
Specialised travel clinic	112 (15.0)
Other^d^	100 (13.4)

^a^Includes non-binary/gender diverse, not listed or prefer not to say

^b^Includes family business, homemaker, student, volunteer

^c^Respondents were able to select more than one option

^d^Includes Different GP & Other.

^e^Includes countries in Oceania outside of Australia

^*^Missing data (country of birth n = 2, age n = 1, private health insurance n = 1)

**Figure 2:**
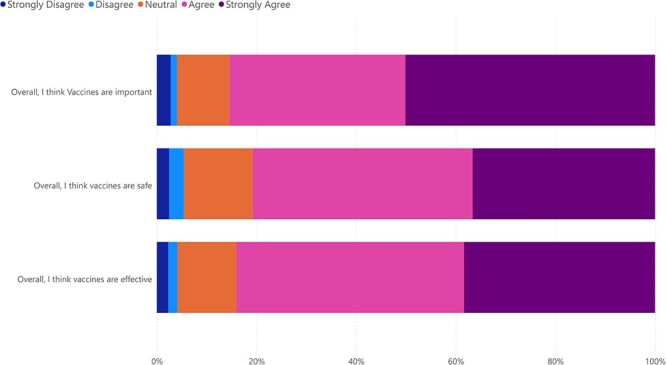
Responses to Vaccine Confidence Index questions regarding the importance, safety and effectiveness of vaccines.

Half (n = 1223, 50%) strongly agreed that vaccines were important for their health, but less than half strongly agreed that vaccines were safe (37%) and effective (38%; Figure 2). Only 3% or fewer strongly disagreed with these statements. Most (n = 873, 71%) had received an influenza vaccine in the past 12 months, typically at a primary care clinic (n = 682, 78%) or local pharmacy (n = 139, 16%; Table S2). Top sources of vaccine information were GPs (n = 1062, 87%) and the Internet (n = 529, 43%). Most indicated high levels of trust in vaccine information from health professionals (n = 776, 63%) and little-to-no trust in information from social media (n = 1046, 85%), religious leaders (n = 1003, 82%) and travel agents (n = 744, 61%; Table S3).

Multivariable models adjusted for age, gender, travel count, travel to higher-risk regions and VFR travel indicated that for every ten-year increase in age, there was an approximately 18% increase in the odds of past uptake of pre-travel vaccination (OR 1.17 [95% CI 1.08–1.27], p < 0.001; Table S4). Travel to higher-risk destinations (2.92 [2.17–3.93], p < 0.001) also increased the odds of past pre-travel vaccine uptake, whereas visiting friends and relatives (VFR) travellers were less likely to have received past pre-travel vaccines (0.74 [0.56–0.97], p = 0.028).

**Table 2:** Demographic and travel predictors of wanting to receive pre-travel vaccination against diseases outlined in hypothetical scenarios

	Scenario 1 (Disease X)		Scenario 2 (Disease Y)	
Characteristic	**Odds ratio (95% CI)** ^**a**^	p-value	**Odds ratio (95% CI)** ^**a**^	p-value
Age	1.01 (0.91–1.11)	0.897	0.98 (0.90–1.07)	0.634
Gender	0.96 (0.70–1.32)	0.811	1.12 (0.86–1.46)	0.386
Pre-travel vaccination	2.60 (1.91–3.56)	<0.001	1.89 (1.45–2.47)	<0.001
Number of past overseas trips	0.98 (0.86–1.13)	0.825	1.03 (0.92–1.16)	0.598
Visting friends and relatives (VFR) travel	0.72 (0.52–1.00)	0.049	0.75 (0.57–0.99)	0.041
Agree that vaccines are safe	7.18 (5.07–10.18)	<0.001	3.64 (2.63–5.03)	<0.001

^a^Obtained through logistic regression model including the following variables: age (categorical, ten-year age groups), gender (binary), pre-travel vaccination (binary), number of past overseas trips (categorical), VFR travel (binary), agreement that vaccines are safe (binary)

Hypothetical scenario responses indicated that a greater proportion would want to receive pre-travel vaccination against Disease X (n = 948, 78%) compared to Disease Y (n = 813, 66%; Table S5). Similarly, more respondents thought their family and friends would want them to be vaccinated against Disease X compared to Disease Y (82 vs. 74%) and indicated it would be moderately or very easy to pay for vaccination against Disease X compared to Disease Y (79 vs. 56%; Table S6). In multivariable models, predictors of wanting to receive pre-travel vaccination against hypothetical diseases included past pre-travel vaccination (Disease X: OR 2.60 [95% CI 1.91–3.56] p < 0.001; Disease Y: OR 1.89 [1.45–2.47] p < 0.001) and agreement that vaccines are safe (Disease X: OR 7.18 [5.07–10.18], p < 0.001; Disease Y: OR 3.64 [2.63–5.03], p < 0.001; Table 2). In contrast, past VFR travel was predictive of not wanting to receive pre-travel vaccination (Disease X: OR 0.72 [0.52–1.00], p = 0.049; Disease Y: OR 0.75 [0.57–0.99], p = 0.041). For both scenarios, VFR travellers were more likely to indicate that family and friends would not want them to get vaccinated (Disease X: OR 0.61 [0.43–0.87], p = 0.006); Disease Y: OR 0.72 [0.54–0.96], p = 0.023; Table S7). In contrast, older age (Disease X only: OR 1.14 [1.03–1.27], p = 0.013), past pre-travel vaccination (Disease X: OR 2.50 [1.78–3.51], p < 0.001; Disease Y: OR 1.83 [1.38–2.43], p < 0.001) and agreement that vaccines are safe (Disease X: OR 5.81 [4.05–8.35], p < 0.001; Disease Y: OR 2.95 [2.12–4.09], p < 0.001) were predictive of thinking that family and friends would support vaccination. Similarly, older age, agreement that vaccines were safe and a greater number of overseas trips were predictive of thinking it would be easy to pay for vaccines (Table S8). Those reporting past VFR travel were less likely than other travellers to consider it moderately/very easy to pay for vaccination against Disease Y (OR 0.67 [0.51–0.87], p = 0.002; Table S8).

Information considered very important to vaccine decision making included protective efficacy of the vaccine (n = 796; 65%), health risks of disease (n = 789, 65%), vaccine side effects (n = 724, 59%), the chance of getting the disease if unvaccinated (n = 721, 59%) and availability of treatment (n = 707, 58%; Figure S2). Two thirds (63%) were interested in using a decision aid, with most preferring to do so with a trusted health professional; the most preferred format was web-based (59%; Table S9).

Free-text responses to the question “What additional information would be useful in a vaccine decision aid” were provided by 295 respondents (24%). Thematic analysis yielded four key themes: practical considerations, destination-based disease risk and vaccine requirements, vaccine safety and efficacy considerations, and details of vaccine testing, manufacturing and components (Table 3). Ninety-nine (8%) respondents provided free-text responses to the question “is there anything else you’d like us to know”, yielding three key themes: support for travel vaccine decision aids, desire for health professional input/endorsement, and format and delivery suggestions (Table S10).

## Discussion

Our study evaluated information needs and attitudes towards pre-travel vaccines, drivers of vaccine uptake, and levels of vaccine confidence amongst a nationally representative cross-sectional survey of Australians.

A novel study element was our use of the WHO’s BeSD framework to evaluate barriers and enablers to pre-travel vaccine uptake in relation to two disease-vaccine hypotheticals with features similar to influenza (Disease X) and yellow fever (Disease Y). We found that past pre-travel vaccination and confidence in vaccine safety were predictive of wanting to receive vaccination against both diseases, and (along with greater travel experience) were also predictive of finding it easy to pay for vaccines. In contrast, past VFR travel was predictive of not wanting to get vaccinated and lower likelihood of finding it easy to pay for vaccination against disease Y (the more expensive vaccine).

These findings indicate that cost perceptions influence pre-travel vaccine acceptance. While a previous systematic review reported that cost was generally not a significant barrier to seeking pre-travel health advice,^2^ cost has been identified as an important driver of vaccine acceptance in both discrete choice experiment studies^19^^,^^20^ and qualitative studies of VFR travellers.^4^^,^^21^ Our findings also suggest that social norms (e.g. family and friends’ vaccine beliefs and uptake) and health worker recommendations play an important role in motivating pre-travel vaccine uptake, aligning with recent findings from COVID-19 vaccine research.^22^^,^^23^ Determinants of pre-travel vaccine decision-making are clearly multifactorial, and it is likely that certain factors may be stronger barriers or enablers for some individuals than others. Health professionals should be mindful of this when having vaccine-related discussions with their patients.

**Table 3:** Additional information respondents would find useful in a decision aid: themes, sub-themes and illustrative quotes from analysis of free-text responses (n = 295)

Theme	Subtheme(s)	Illustrative quotation(s) (sex, age group and State/Territory of residence of respondent)
Practical considerations	Timing of vaccine in relation to travel	‘How long before travelling I would need the course of vaccines’ (Male, 70–79, VIC) ‘If more than one dose required, what is the timeframe between doses?’ (Female, 60–69, SA)
	Who can give the vaccine (provider type)	‘Who can administer the vaccination, i.e. GP, pharmacist, nurse’ (Male, 70–79, WA)
	Availability and cost	‘How/where to get the vaccine for the lowest cost’ (Female, 40–49, SA)
Destination-based risk and recommendations/requirements	Estimates of risk at traveller’s destination	Incidence of the disease in the destination (Male, 80–89, NSW) ‘Info about the statistics of the disease past & present in the country I am going to’ (Female, 50–59, NSW)
	Destination-based vaccine recommendations or requirements	‘If mandatory or recommended by country [of travel]’ (Female, 60–69, VIC)
Vaccine safety and efficacy considerations	Vaccine contraindications and precautions	‘If it is ok to get vaccinated with cancer treatments’ (Female, 70–79, VIC) ‘Would it interfere with other prescribed medications you take?’ (Female, 50–59, NSW)
	Evidence that the vaccine works (and to what extent)	‘Results of effectiveness or trial results’ (Female, 70–79, ACT) ‘Relative proof that the vaccine works, clinical evidence’ (Male, 60–69, QLD)
	Information about adverse events, including long-term and serious outcomes	‘If there have been any deaths as a direct result of the vaccine’ (Female, 40–49, QLD) ‘All known side or aftereffects both immediate and the long-term’ (Male, 50–59, NSW)
	Data comparing outcomes in vaccinated vs. unvaccinated individuals	‘How much time a [unvaccinated] person would spend in hospital compared to a person who has been vaccinated.’ (Male, 80–89, QLD) ‘What % of Australian travellers have the vaccine and how many that did not acquired the disease’ (Male, 60–69, SA)
Details of vaccine testing, manufacturing and components	Ethical and religious considerations	‘Is it halal or not?’ (Male, 30–39, VIC) ‘If the vaccine had animal products in them might influence my decision to have it’ (Female, 60–69, ACT)
	Information on vaccine manufacturer and testing	‘Who made it, and where it was made’ (Female, 50–59, QLD) ‘The vaccine must have gone through full and rigorous testing over time and not be provisionally approved or a rushed emergency use only approval’ (Male, 60–69, SA)

Our study identified increasing age as a predictor of past pre-travel vaccine uptake but not of pre-travel vaccination against hypothetical diseases. Previous studies have shown differing effects of age on pre-travel vaccination, with some showing improved uptake amongst younger travellers and others showing improved uptake amongst older travellers.^2^ A recent Spanish study found that more than one third of travellers aged over 60 travelling to yellow-fever risk areas rejected vaccination.^24^ This underscores the importance of understanding and addressing the vaccine information needs and concerns of older travellers, especially as the age of travel populations continues to rise.^25^^,^^26^

Our study found that VFR travellers are less likely to seek and/or receive pre-travel vaccines compared to other travellers, consistent with previous studies.^27^^,^^28^ VFR travellers are recognised to be at a disproportionately high risk of travel-associated disease acquisition and subsequent importation, but face barriers in accessing pre-travel health services and are more likely to decline vaccination recommendations than other travellers.^4^^,^^29^^,^^30^ Culturally safe care provision and resources that improve awareness of travel-related VPDs are needed to improve access to and uptake of pre-travel vaccination amongst this traveller sub-group.^4^^,^^31^

A large proportion of participants were experienced travellers, with almost two-thirds having completed over five past overseas trips. Three of respondents’ most common reasons for not receiving pre-travel vaccination were low risk perception, travel to a familiar destination and belief they were already up-to-date with vaccines. These findings lend support to the idea of conducting a multi-trip risk assessment when travellers first present for pre-travel advice that considers the cumulative risks of current and future trips and views vaccines, particularly those providing a longer duration of protection, as an investment for future health, especially amongst younger travellers.^32^ It also suggests an important role for opportunistic pre-travel discussions and vaccination in frequent traveller groups such as business and VFR travellers, particularly given that these groups are more likely to present for consultation last minute and defer vaccination.^33^

It is clear from our findings that primary care practitioners are an important source of pre-travel advice and vaccination. A 2021 survey of US primary care practitioners found a troubling proportion lacked high levels of vaccine confidence.^34^ This highlights the need to investigate whether health professionals’ vaccine confidence influences their vaccine recommendations, given the importance of such recommendations to patients’ vaccine uptake and known associations between the quality of pre-travel advice and patient adherence with preventive measures.^2^ Notably, 25% of our respondents indicated they would see a pharmacist for vaccine information and more than 10% had received influenza vaccine at their community pharmacy in the past 12 months. With programs enabling trained pharmacists to prescribe some travel vaccines already established in three Australian jurisdictions,^(35–37)^ Australian pharmacists are likely to play an expanded role in travel vaccination in the future, as has occurred in countries including the USA, Canada and UK.^38^ This highlights the importance of appropriate vaccine provider training programs and evidence-based resources that support patients’ pre-travel vaccine decision making.

Vaccine confidence reflects trust in vaccines, vaccine providers, vaccine development and regulation.^39^^,^^40^ Empirical data suggests that vaccine confidence is a key determinant of vaccine acceptance, with a study from 149 countries between 2015–2019 correlating an increasing trend of vaccine delays or refusals with reducing confidence in vaccines.^13^ We found lower rates of vaccine confidence across all three domains of the VCI (importance, safety and effectiveness) compared to studies surveying representative Australian cohorts in 2015, 2018 and early 2021^(13,41)^ (Table S11), which supports findings from other countries since the COVID-19 pandemic’s onset.^42^ A 2022 study analysing data from > 880 000 social media comments and an online survey of > 1000 German travellers found that reluctance to receive vaccination was most commonly related to low perceived risk of disease exposure, cost of vaccination, fear of vaccine side-effects and number of vaccines.^43^ Recent studies around patient-facing vaccine information indicate that the most effective messages for reducing vaccine hesitancy are those that highlight the personal benefits of vaccines (e.g. prevention of serious illness).^44^ Amongst our survey participants, most felt it was very important to have information on a range of disease and vaccine attributes when making vaccine-related decisions. Additionally, themes arising from free-text responses indicated a desire for evidence-based information on vaccine safety and efficacy, destination-based data on risks and requirements and (where available) data comparing outcomes in vaccinated and unvaccinated individuals. This underscores the need for evidence-based, unbiased information resources to support vaccine decision-making. Further research is needed to understand optimal design and delivery methods for vaccine decision support tools that cater to different levels of education and literacy and to people from culturally and linguistically diverse groups. Co-design research methods that involve consumers and health professionals in tool design may support the development of tools that cater to different needs and resonate with end users.

### Strengths and limitations

This is one of few surveys that has evaluated pre-travel vaccine attitudes, behaviours and information needs. Amongst eligible participants, the survey response rate was very high (92%), which may reflect the use of a consumer panel. Consumer panels can be subject to bias and may not be fully representative of the general population; panel members tend to have greater internet access and higher socio-economic status compared to the general population, particularly amongst older age groups.^14^ While we acknowledge that this survey in the English language may have resulted in under-representation of some key groups, such as members of culturally and linguistically diverse (CALD) communities, we note that one-third of our survey participants were born overseas and almost one-third reported a history of VFR travel. The proportion of business travellers in our survey was small (4%), but this is likely explained by the relatively large proportion of retirees (46%) within our cohort. Inclusion of a large cohort of adults aged > 65 in our survey is a strength, as this is an important traveller group often under-represented in travel medicine studies.

## Conclusion

Our study highlighted information needs and attitudes towards pre-travel vaccines, drivers of vaccine uptake, and trends in vaccine confidence amongst Australians in December 2022. While health professionals remain integral to supporting pre-travel vaccine decision-making, the high proportion of individuals already using the internet for travel-related health information and existing challenges around communicating risks and ensuring information recall across a broad range of health topics in a pre-travel consultation^2^^,^^3^ indicate a growing need for reliable, accurate and engaging materials that can supplement pre-travel consultations and aid informed vaccine decision-making. Findings from this study can be used to improve pre-travel health advice provision and inform development of patient-facing resources, such as decision aids, to support travellers to make informed pre-travel vaccine decisions.

## Supplementary data

Supplementary data are available at *JTM* online.

## Data availability statement

The data that support the findings of this study are available upon request from the corresponding author, SM, upon reasonable request. Supplementary data is available in supplementary files.

## Funding

This work was supported by an International Society of Travel Medicine Research Grant (grant number 369083185). SLM (grant number 2017229) and AC (grant number 1194678) are supported by National Health and Medical Research Council (NHMRC) Investigator Grants and KL (grant number 1155005) is supported by a NHMRC Senior Research Fellowship. Funding agencies were not involved in study design, the collection, analysis, and interpretation of data or the writing of the report.

## Authors’ contribution

Sarah McGuinness (Conceptualisation-Lead, Funding acquisition-Equal, Methodology-Equal, Investigation-Equal, Data curation-Equal, Formal analysis-Equal, Resources-Equal, Writing—original draft-Lead, Writing—review & editing-Equal, Project administration-Equal, Validation-Equal, Visualisation-Equal), Owen Eades (Conceptualisation-Supporting, Methodology-Equal, Investigation-Equal, Data curation-Equal, Formal analysis-Equal, Writing—original draft-Supporting, Writing—review & editing-Equal, Project administration-Equal, Validation-Equal, Visualisation-Equal), Allen Cheng, (Conceptualisation-Supporting, Methodology-Equal, Investigation-Supporting, Supervision-Supporting, Writing—review & editing-Equal), Holly Seale (Conceptualisation-Supporting, Methodology-Equal, Investigation-Equal, Supervision-Supporting, Writing—review & editing-Equal) and Karin Leder (Conceptualisation-Supporting, Funding acquisition-Equal, Methodology-Equal, Investigation-Supporting, Resources-Equal, Supervision-Lead, Writing—review & editing-Equal).

## Conflict of interest

HS has received funding from vaccine companies including Sanofi Pasteur, Moderna and CSL Seqirus. This funding was not used for this project. The other authors have no conflicts of interest to declare.

## Supplementary Material

Supplementary_Appendix-TRAVAID_Manuscript_taad056

Supplementary_file_TRAVAID_survey_questions_taad056
