# Supplementary material for: Pre-travel vaccine information needs, attitudes, drivers of uptake and the role for decision aids in travel medicine
Source: J Travel Med. 2023 Apr 19;30(4):taad056. doi: 10.1093/jtm/taad056 (PMC10289516; doi:10.1093/jtm/taad056)
Supplement: Supplementary_Appendix-TRAVAID_Manuscript_taad056 [file supplementary_appendix-travaid_manuscript_taad056.docx]

**Supplementary materials**

Table of Contents

[**Table S1:** Disease and vaccine characteristics for each of the hypothetical scenarios 2](#_Toc131094645)

[**Table S2:** Recent influenza vaccine practices 2](#_Toc131094646)

[**Table S3:** Use of and Trust in different sources of vaccine information 3](#_Toc131094647)

[**Table S4:** Associations between past pre-travel vaccination amongst 1161 participants with a history of overseas travel 3](#_Toc131094648)

[**Table S5:** Univariate analysis of associations between demographic and travel characteristics and wanting to receive pre-travel vaccination against hypothetical diseases 4](#_Toc131094649)

[**Table S6**: Adapted BeSD hypothetical scenario-based responses among those who did and did not want to be vaccinated for each scenario 5](#_Toc131094650)

[**Table S7:** Demographic and travel predictors of thinking that most close family and friends would want you to be vaccinated against diseases outlined in hypothetical scenarios 5](#_Toc131094651)

[**Table S8:** Demographic and travel predictors of considering it moderately or very easy to pay for vaccines against diseases outlined in hypothetical scenarios 6](#_Toc131094652)

[**Table S9:** Interest in using a decision aid 6](#_Toc131094653)

[**Table S10.** Additional information respondents wanted to communicate: themes, sub-themes and illustrative quotes from analysis of free-text responses, n=99 7](#_Toc131094654)

[**Table S11:** Comparison of responses to Vaccine Confidence Index questions with other population-based Australian samples 8](#_Toc131094655)

[**Figure S1.** Participant flow diagram 9](#_Toc131094656)

[**Figure S2**. Participants perceptions’ of how important information on certain disease and vaccine attributes would be to their decision making 10](#_Toc131094657)

[**References** 11](#_Toc131094658)

## **Table S1:** Disease and vaccine characteristics for each of the hypothetical scenarios

| **Characteristic** | **Disease X (Scenario 1)** | **Disease Y (Scenario 2)** |
| --- | --- | --- |
| **Route of transmission** | Person-to-person | Vector-borne (mosquitoes) |
| **Present in Australia** | No | No |
| **Risk of acquisition during 2-week trip** | 1 in 100 (1%) | 1 in 100,000 (0.001%) |
| **Chance of mild illness (if infected)** | 99 in 100 (99%) | 1 in 2 (50%) |
| **Chance of severe illness (if infected)** | 1 in 100 (1%) | 1 in 2 (50%) |
| **Risk of death amongst those with severe illness** | 1 in 100 (1%) | 1 in 2 (50%) |
| **Vaccine efficacy** | ~50% | >99.9% |
| **Duration of protection from vaccine** | 6 months | Lifelong |
| **Vaccine side effects** | Mild only (e.g. injection site reaction) | Generally safe, but death reported in ~1 in 1,000,000 doses (0.0001%) |
| **Vaccine cost** | $20 AUD | $200 |

AUD = Australian dollars

## **Table S2:** Recent influenza vaccine practices

| **Received influenza vaccine in past 12 months (n=1223)** | |
| --- | --- |
| Yes | 873 (71.4) |
| No | 339 (27.7) |
| Not sure | 11 (0.9) |
| **Where was influenza vaccine given?** | |
| GP / community health centre | 682 (78.1) |
| Hospital / specialist’s rooms | 16 (1.8) |
| Pharmacy / chemist | 139 (15.9) |
| Workplace | 35 (4.0) |
| Other | 1 (<1) |

## **Table S3:** Use of and Trust in different sources of vaccine information

| **Trust in information from:** |  |  |  |
| --- | --- | --- | --- |
|  | **Little to no trust**  **n (%)** | **Moderate trust**  **n (%)** | **High trust**  **n (%)** |
| **Australian Government** | 280 (22.9) | 462 (37.8) | 481 (39.3) |
| **Health professionals** | 127 (10.4) | 320 (26.2) | 776 (63. 5) |
| **Community health centres** | 169 (13.8) | 437 (35.7) | 617 (50.5) |
| **Travel medicine clinics** | 208 (17.0) | 462 (37.8) | 553 (45.2) |
| **NGOs (research institutes)** | 397 (32.5) | 568 (46.4) | 258 (21.1) |
| **Social media** | 1,046 (85.5) | 136 (11.1) | 41 (3.4) |
| **Travel agents** | 744 (60.8) | 382 (31.2) | 97 (7.9) |
| **Family and friends** | 611 (50.0) | 463 (37.9) | 149 (12.2) |
| **Community leaders** | 891 (72.9) | 287 (23.5) | 45 (3.7) |
| **Religious leaders** | 1,003 (82.0) | 172 (14.1) | 48 (3.9) |
|  |  |  |  |
| **Sources of vaccine information:** | | |  |
|  | | | **Use**  **n (%)** |
| **The internet** | | | 529 (43.3) |
| **General Practitioner (GP)** | | | 1062 (86.8) |
| **Travel medicine specialist or clinic** | | | 367 (30.0) |
| **Pharmacist** | | | 311 (25.4) |
| **Travel agent** | | | 244 (20.0) |
| **Family and/or friends** | | | 140 (11.5) |
| **Employer** | | | 24 (2.0) |
| **Community leader** | | | 13 (1.1) |
| **Religious leaders** | | | 9 (0.7) |
| **Other** | | | 37 (3.0) |

## **Table S4:** Associations between past pre-travel vaccination amongst 1161 participants with a history of overseas travel

| **Characteristic** | **Odds ratio (95% CI)** | **p-value** |
| --- | --- | --- |
| **Age** | **1.17 (1.08 – 1.27)** | **<0.001** |
| **Gender** | 1.21 (0.94 – 1.57) | 0.141 |
| **Number of past overseas trips** | 1.11 (0.99 – 1.25) | 0.077 |
| **Travel to higher-risk regions**^b^ | **2.92 (2.17 – 3.93)** | **<0.001** |
| **VFR travel** | **0.74 (0.56 – 0.97)** | **0.028** |

^a^Obtained through a logistic regression model including the following variables: age (categorical, 10-year age groups), gender (binary), travel count (categorical), high-risk region (binary), VFR travel (binary)

^b^ Past travel to Asia (including South Asia), Africa, Central/South America or the Middle East

## **Table S5:** Univariate analysis of associations between demographic and travel characteristics and wanting to receive pre-travel vaccination against hypothetical diseases

|  | **Scenario 1 (Disease X)** | | | **Scenario 2 (Disease Y)** | | |
| --- | --- | --- | --- | --- | --- | --- |
| **Wanting to receive vaccination** | **Yes n=948** | **No n=275** | **p-value** | **Yes n=813** | **No n=410** | **p-value** |
| **Age, years** | **n (%)** | **n (%)** |  | **n (%)** | **n (%)** |  |
| **10-19**  **20-29**  **30-39**  **40-49**  **50-59**  **60-69**  **70-79**  **80-89**  **90-99** | 2 (0.2)  47 (5.0)  90 (9.5)  104 (11.0)  109 (11.5)  239 (25.2)  292 (30.8)  62 (6.6)  2 (0.2) | 1 (0.4)  16 (5.8)  36 (13.1)  46 (16.7)  51 (18.6)  58 (21.1)  53 (19.3)  13 (4.7)  1 (0.4) | **<0.001** | 2 (0.3)  36 (4.4)  86 (10.6)  93 (11.5)  96 (11.8)  198 (24.4)  249 (30.7)  51 (6.3)  1 (0.1) | 1 (0.2)  27 (6.6)  40 (9.8)  57 (13.9)  64 (15.6)  99 (24.2)  96 (23.4)  24 (5.9)  2 (0.5) | 0.093 |
| **Gender** |  |  |  |  |  |  |
| **Female** | 477 (50.4) | 151 (55.1) |  | 403 (49.7) | 225 (54.9) |  |
| **Male** | 469 (49.6) | 123 (44.9) | 0.172 | 407 (50.3) | 185 (45.1) | 0.091 |
| **Country of birth** | | | | | | |
| **Australia** | 640 (67.6) | 173 (63.1) |  | 553 (68.1) | 260 (63.6) |  |
| **Overseas** | 307 (32.4) | 101 (36.9) | 0.170 | 259 (31.9) | 149 (36.4) | 0.113 |
| **Language spoken at home** | | | | | | |
| **English only** | 823 (86.8) | 218 (79.3) |  | 705 (86.7) | 336 (81.9) |  |
| **Other** | 125 (13.2) | 57 (20.7) | **0.002** | 108 (13.3) | 74 (18.1) | **0.027** |
| **Employment status** | | | | | | |
| **Unpaid/unemployed** | 97 (10.2) | 49 (17.8) |  | 90 (11.1) | 56 (13.7) |  |
| **Employed** | 377 (39.8) | 136 (49.5) |  | 329 (40.5) | 184 (44.9) |  |
| **Retirees** | 474 (50.0) | 90 (32.7) | **<0.001** | 394 (48.5) | 170 (41.5) | 0.058 |
| **Private health insurance** | | | | | | |
| **Yes** | 584 (61.7) | 135 (49.1) |  | 502 (61.8) | 217 (52.9) |  |
| **No** | 363 (38.3) | 140 (50.9) | **<0.001** | 310 (38.2) | 193 (47.1) | **0.003** |
| **Number of past overseas trips** | | | | | | |
| **1 or 2** | 160 (17.8) | 64 (24.5) |  | 135 (17.6) | 89 (22.7) |  |
| **3 or 4** | 159 (17.7) | 35 (13.4) |  | 125 (16.3) | 69 () 17.6 |  |
| **5 to 9** | 211 (23.4) | 68 (26.1) |  | 195 (25.4) | 84 (21.4) |  |
| **10 or more** | 370 (41.1) | 94 (36.0) | **0.031** | 314 (40.8) | 150 (38.3) | 0.114 |
| **Travel to higher-risk destinations** | | | | | | |
| **Higher risk** | 236 (26.2) | 74 (28.4) |  | 567 (73.7) | 284 (72.5) |  |
| **Lower risk** | 664 (73.8) | 187 (71.6) | 0.493 | 202 (26.3) | 108 (27.5) | 0.640 |
| **Pre-travel vaccination** | | | | | | |
| **Yes** | 634 (70.4) | 114 (43.7) |  | 543 (70.6) | 205 (52.3) |  |
| **No** | 266 (29.6) | 147 (56.3) | **<0.001** | 226 (29.4) | 187 (47.7) | **<0.001** |
| **Visiting friends and relatives (VFR) travel** | | | | | | |
| **VFR** | 261 (29.0) | 97 (37.2) |  | 218 (28.4) | 140 (35.70 |  |
| **Other*** | 639 (71.0) | 164 (62.8) | **0.012** | 551 (71.6) | 252 (64.3) | **0.010** |
| **Time since last overseas trip (years)** | | | | | | |
| **Less than 5** | 570 (63.3) | 150 (57.5) |  | 480 (62.4) | 240 (61.2) |  |
| **5 or more** | 330 (36.7) | 111 (42.5) | 0.086 | 289 (37.6) | 152 (38.8) | 0.692 |
| **Agreement that vaccines are important for health** | | | | | | |
| **Agree** | 885 (93.4) | 160 (58.2) |  | 752 (92.5) | 293 (71.5) |  |
| **Disagree** | 63 (6.7) | 115 (41.8) | **<0.001** | 61 (7.5) | 117 (28.5) | **<0.001** |
| **Agreement that vaccines are safe** | | | | | | |
| **Agree** | 848 (89.5) | 140 (50.9) |  | 718 (88.3) | 270 (65.8) |  |
| **Disagree** | 100 (10.5) | 135 (49.1) | **<0.001** | 95 (11.7) | 140 (34.2) | **<0.001** |
| **Agreement that vaccines are effective** | | | | | | |
| **Agree** | 864 (91.1) | 164 (59.6) |  | 82 (10.1) | 113 (27.6) |  |
| **Disagree** | 84 (8.9) | 111 (40.4) | **<0.001** | 731 (89.9) | 297 (72.4) | **<0.001** |

## **Table S6**: Adapted BeSD hypothetical scenario-based responses among those who did and did not want to be vaccinated for each scenario

|  | **Disease X (Scenario 1)** | | | | **Disease Y (Scenario 2)** | | | |
| --- | --- | --- | --- | --- | --- | --- | --- | --- |
|  | **No / Not sure** | **Yes** | **Total** | **P-value** | **No / Not sure** | **Yes** | **Total** | **P-value** |
| **Do you think most of your close family and friends would want you to get vaccinated against Disease X/Y before you travel?** | | | | | | | | |
| **Yes** | 92 (33.5%) | 917 (96.7%) | 1009 (82.5%) |  | 121 (29.5%) | 785 (96.6%) | 906 (74.1%) |  |
| **No** | 183 (66.5%) | 31 (3.3%) | 214 (17.5%) |  | 289 (70.5%) | 28 (3.4%) | 317 (25.9%) |  |
| **Total** | 275 (100%) | 948 (100%) | 1223 (100%) | **<0.001** | 410 (100%) | 813 (100%) | 1223 (100%) | **<0.001** |
| **How easy would it be for you to pay for the vaccine against Disease X/Y?** | | | | | | | | |
| **Moderately / Very easy** | 167 (60.7%) | 797 (84.1%) | 964 (78.8%) |  | 154 (37.6%) | 525 (65.6%) | 679 (55.5%) |  |
| **Not at all / A little easy** | 108 (39.3%) | 151 (15.9%) | 259 (21.2%) |  | 256 (62.4%) | 288 (35.4%) | 544 (44.5%) |  |
| **Total** | 275 (100%) | 948 (100%) | 1223 (100%) | **<0.001** | 410 (100%) | 813 (100%) | 1223 (100%) | **<0.001** |

## **Table S7:** Demographic and travel predictors of thinking that most close family and friends would want you to be vaccinated against diseases outlined in hypothetical scenarios

|  | **Scenario 1 (Disease X)** | | **Scenario 2 (Disease Y)** | |
| --- | --- | --- | --- | --- |
| **Characteristic** | **Odds ratio (95% CI)^a^** | **p-value** | **Odds ratio (95% CI)^a^** | **p-value** |
| **Age** | **1.14 (1.03 – 1.27)** | **0.013** | 1.02 (0.94 – 1.12) | 0.602 |
| **Gender** | 1.01 (0.72 – 1.42) | 0.959 | 1.06 (0.81 – 1.41) | 0.660 |
| **Pre-travel vaccination** | **2.50 (1.78 – 3.51)** | **<0.001** | **1.83 (1.38 – 2.43)** | **<0.001** |
| **Number of past overseas trips** | 0.84 (0.72 – 0.97) | 0.022 | 0.92 (0.81 – 1.04) | 0.177 |
| **Visting friends and relatives (VFR) travel** | **0.61 (0.43 – 0.87)** | **0.006** | **0.72 (0.54 – 0.96)** | **0.023** |
| **Agree that vaccines are safe** | **5.81 (4.05 – 8.35)** | **<0.001** | **2.95 (2.12 – 4.09)** | **<0.001** |

^a^ Obtained through logistic regression model including the following variables: age (categorical, 10-year age groups), gender (binary), pre-travel vaccination (binary), number of past overseas trips (categorical), VFR travel (binary), agreement that vaccines are safe (binary)

## **Table S8:** Demographic and travel predictors of considering it moderately or very easy to pay for vaccines against diseases outlined in hypothetical scenarios

|  | **Scenario 1 (Disease X)** | | **Scenario 2 (Disease Y)** | |
| --- | --- | --- | --- | --- |
| **Characteristic** | **Odds ratio (95% CI)^a^** | **p-value** | **Odds ratio (95% CI)^a^** | **p-value** |
| **Age** | **1.22 (1.11 – 1.34)** | **<0.001** | **1.10 (1.02 – 1.19)** | **0.013** |
| **Gender** | 1.03 (0.76 – 1.41) | 0.837 | **1.35 (1.05 – 1.72)** | **0.018** |
| **Pre-travel vaccination** | 1.05 (0.76 – 1.44) | 0.784 | 1.16 (0.89 – 1.50) | 0.265 |
| **Number of past overseas trips** | **1.36 (1.19 – 1.55)** | **<0.001** | **1.17(1.05 – 1.30)** | **0.005** |
| **Visting friends and relatives (VFR) travel** | 0.85 (0.61 – 1.17) | 0.316 | **0.67 (0.51 – 0.87)** | **0.002** |
| **Agree that vaccines are safe** | **3.47 (2.46 – 4.89)** | **<0.001** | **2.25 (1.63 – 3.10)** | **<0.001** |

^a^ Obtained through logistic regression model including the following variables: age (categorical, 10-year age groups), gender (binary), pre-travel vaccination (binary), number of past overseas trips (categorical), VFR travel (binary), agreement that vaccines are safe (binary)

## **Table S9:** Interest in using a decision aid

| **Proportion who agree/disagree ^a^ they would:** |  |  |
| --- | --- | --- |
|  | **Disagree**  **n (%)** | **Agree**  **n (%)** |
| **Be interested in using a decision aid** | 455 (37.2) | 768 (62.8) |
| **Prefer to use a decision aid alone before seeing a health professional** | 727 (59.4) | 496 (40.6) |
| **Prefer to use a decision aid with family and/or friends before seeing a health professional** | 827 (67.6) | 396 (32.4) |
| **Prefer to use a decision with a trusted health professional** | 381 (31.2) | 842 (68.9) |
|  |  |  |
| **Likelihood of using a decision aid in a:** |  |  |
|  | **Unlikely ^b^**  **n (%)** | **Likely**  **n (%)** |
| **Web-based interactive format** | 499 (40.8) | 724 (59.2) |
| **Video-based format** | 760 (62.1) | 463 (37.9) |
| **Paper-based format** | 710 (58.1) | 513 (41.9) |
| **PDF format** | 614 (50.2) | 609 (49.8) |

**^a^** obtained using a 5-point Likert scale from strongly disagree to strongly agree. Disagree included responses from strongly disagree to neutral, agree included responses from agree to strongly agree.

^b^ Unlikely included neutral responses

## **Table S10.** Additional information respondents wanted to communicate: themes, sub-themes and illustrative quotes from analysis of free-text responses, n=99

| **Theme** | **Illustrative quotation** |
| --- | --- |
| **Desire for health professional input / endorsement** | “I would like my doctor to look it over and advise me on what I should do.” (Female, 70-79, NSW)  “I think I would still check with my GP even after reading the Travel Aid” (Female, 60-69, QLD)  “Decision ratified by my GP” (Female, 60-69, VIC)  “I would ONLY accept the advice of my regular GP, as he knows my medical history, including what vaccines I’ve already had and how any vaccines may affect or be affected by other medicines I am taking. An online decision aid cannot reliably take these factors into account.” (Male, 70-79, VIC)  “Would be good if the info could be ‘approved’ and recorded in my doctor’s records” (Female, 70-79, NSW)  “I think this service would work well together with GP” (Female, 70-79, VIC) |
| **Support for travel vaccine decision aids** | “It sounds useful, a decision aid would keep people up to date with whatever vaccines they need**.**” (Female, 40-49, VIC)  “This is a great well needed idea” (Female, 50-59, NSW) |
| **Suggestions for decision aid format and/or additional tools** | “Interactive site where I can print the final result would be useful to me.” (Female, 80-89, QLD)  “I prefer the video version as you can watch it several times to make sure you understand the message.” (Male, 80-89, VIC)  “Would also use a smart mobile phone application on a standard platform - eg apple” (Female, 60-69, VIC)  “A chat bot can also help providing education relating to this.” (Male, 50-59, WA) |

## **Table S11:** Comparison of responses to Vaccine Confidence Index questions with other population-based Australian samples

|  | **Strongly Agree, n (%)** | | | | **Strongly Disagree, n (%)** | | | |
| --- | --- | --- | --- | --- | --- | --- | --- | --- |
|  | 2015^a^ | 2018^a^ | 2021 | 2022 (this study) | 2015^a^ | 2018^a^ | 2021 | 2022 (this study) |
| **Considers vaccines important for health** | 900/1190 (76) | 824/988 (83) | 771/1166 (66) | 613/1223 (50) | 19/1190 (2) | 18/988 (2) | 8/1166 (1) | 34/1223 (3) |
| **Considers vaccines safe** | 793/1190 (67) | 602/988 (61) | 691/1166 (59) | 448/1223 (37) | 21/1190 (2) | 33/988 (3) | 9/1166 (1) | 30/1223 (2) |
| **Considers vaccines effective** | 833/1190 (70) | 707/988 (72) | 705/1166 (60) | 469/1223 (38) | 21/1190 (2) | 17/988 (2) | 10/1166 (1) | 28/1223 (2) |

^a^Vaccine confidence index estimates (raw data) for Australian general population in 2015 and 2018 from De Figueiredo et al. (1), supplementary appendix 2.

*Vaccine confidence index estimates for Australians from the general population in February-March of 2021 from Enticott et al. (2).

## **Figure S1.** Participant flow diagram

**
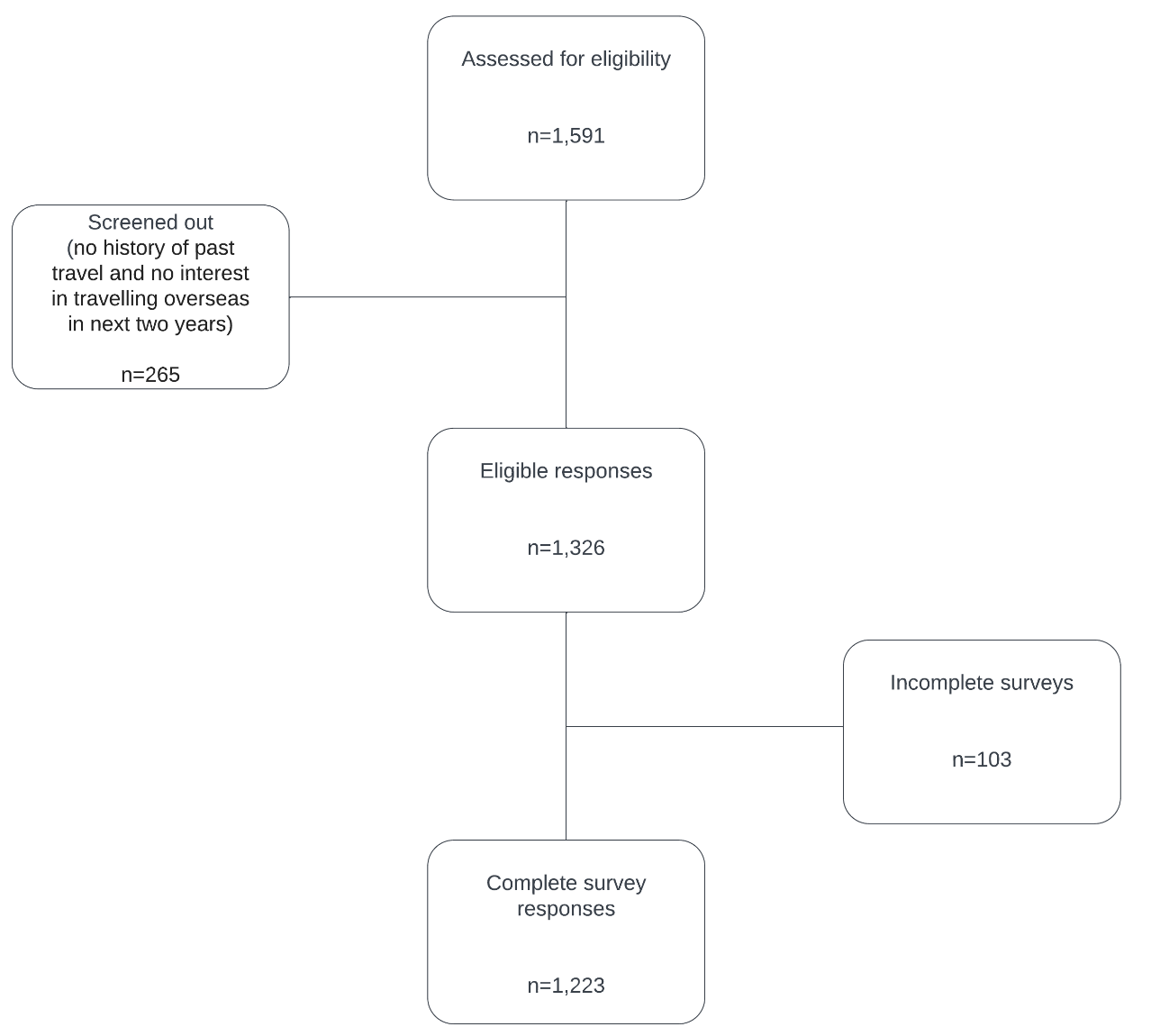
**

## **Figure S2**. Participants perceptions’ of how important information on certain disease and vaccine attributes would be to their decision making


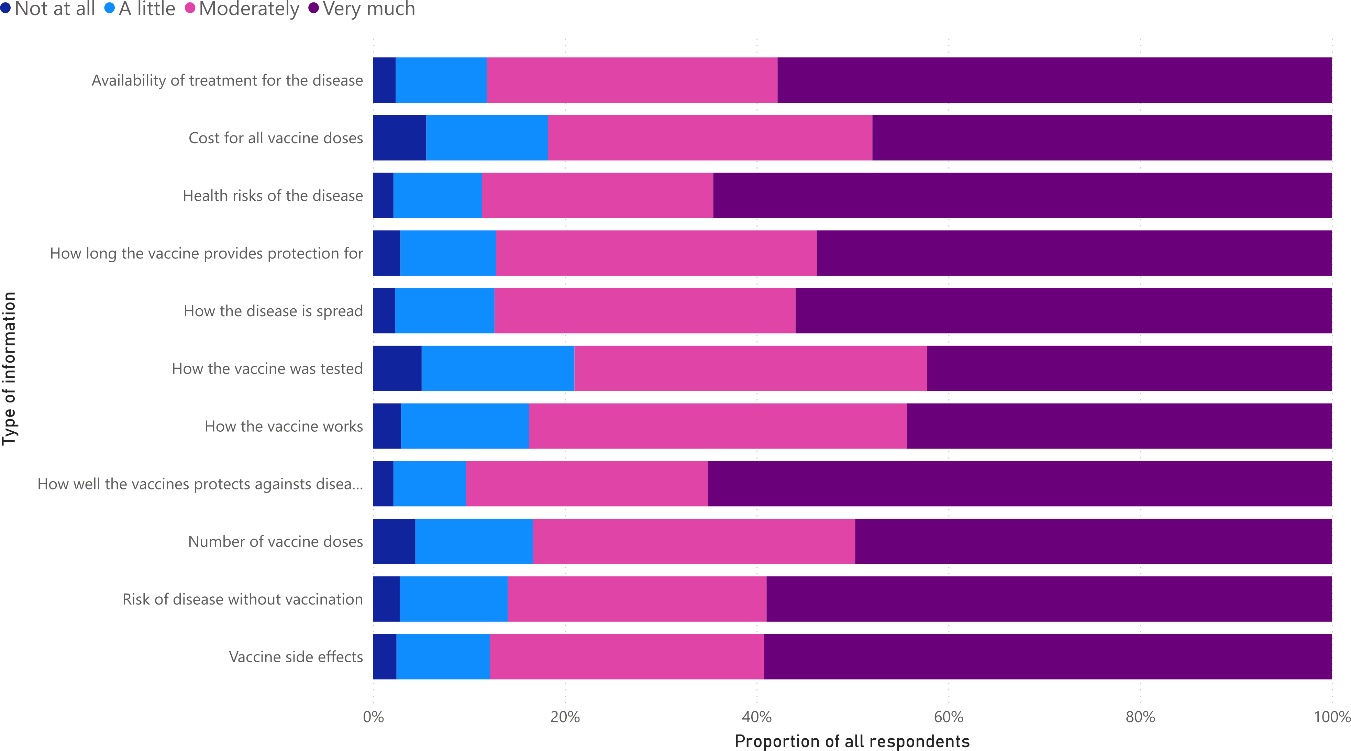


## **References**

1. de Figueiredo A, Simas C, Karafillakis E, et al. Mapping global trends in vaccine confidence and investigating barriers to vaccine uptake: a large-scale retrospective temporal modelling study. The Lancet. 2020;396(10255):898-908.

2. Enticott J, Gill JS, Bacon SL, et al. Attitudes towards vaccines and intention to vaccinate against COVID-19: a cross-sectional analysis—implications for public health communications in Australia. BMJ Open. 2022;12(1):e057127.
