# Supplementary material for: Pre-travel vaccine information needs, attitudes, drivers of uptake and the role for decision aids in travel medicine
Source: J Travel Med. 2023 Apr 19;30(4):taad056. doi: 10.1093/jtm/taad056 (PMC10289516; doi:10.1093/jtm/taad056)
Supplement: Supplementary_file_TRAVAID_survey_questions_taad056 [file supplementary_file_travaid_survey_questions_taad056.pdf]

TRAVAID Online Survey

Thank you for your interest in this study run by Monash University. Please carefully read the instructions below and follow the prompts to begin the survey.

What is this study about?

Overseas travellers often experience travel-related illnesses, some of which can be prevented with vaccines. However, many travellers do not seek or receive vaccines before travelling.

We want to find out what Australians think about travel vaccines, if and where they go for information and advice about them, and what sort of resources would help them to make informed decisions around travel vaccines.

The study is open to anyone aged 18 years and over currently living in Australia who has EITHER travelled overseas in the past OR is intending to travel overseas in the next 2 years.

What does it involve?

We will ask you to complete a short online survey that will take approximately 10-15 minutes. All survey responses are confidential. You can stop at any time if you feel unhappy answering the questions by closing your browser. However, once you have submitted the questionnaire, we will not be able to withdraw your responses as the questionnaire is anonymous

What are the next steps?

Participation in this study is entirely voluntary. The Participant Explanatory Statement has more information about the study.

[Attachment: "TRAVAID - Explanatory statement - online survey_v1.1.03.11.22.pdf"]

If you do not want to participate, please close your browser window.

We appreciate your help with this study!

By clicking the next button below, you consent to be part of this study.

**Part A: Travel experience**<br><br>**This first group of questions asks about your past travel experiences.**	
Have you ever travelled overseas?	◯ Yes<br>◯ No
Thinking back to your most recent overseas trip:	
In what year was this trip?	◯ 2022<br>◯ 2021<br>◯ 2020<br>◯ 2019<br>◯ 2018<br>◯ 2017<br>◯ 2016<br>◯ 2015<br>◯ 2014<br>◯ 2013<br>◯ 2012<br>◯ 2011<br>◯ 2010<br>◯ 2009<br>◯ 2008<br>◯ 2007<br>◯ 2006<br>◯ 2005<br>◯ 2004<br>◯ 2003<br>◯ 2002<br>◯ 2001<br>◯ 2000
In which country did you spend the most time abroad?	◯ China<br>◯ Fiji<br>◯ India<br>◯ Indonesia<br>◯ Japan<br>◯ New Zealand<br>◯ Singapore<br>◯ Thailand<br>◯ UK<br>◯ USA<br>◯ Other country not listed, please specify
Other country not listed, please specify:	__________________________________
What was your main reason for travel?	◯ Tourism (holiday)<br>◯ Visiting friends and relatives<br>◯ Business (e.g. meeting, conference)<br>◯ Education (e.g. study abroad)<br>◯ Other, please specify
Other reason, please specify:	__________________________________
Thinking back to all the overseas trips you have taken in your lifetime:	

How many overseas trips have you taken in total? (select one only)	◯ 1 or 2<br>◯ 3 or 4<br>◯ Between 5 and 10<br>◯ More than 10<br>◯ None
Which of the following regions have you visited (select all that apply):	☐ Asia (excluding South Asia)<br>☐ South Asia (India, Pakistan, Bangladesh, Nepal, Sri Lanka)<br>☐ Africa<br>☐ North America<br>☐ Central and South America<br>☐ Europe<br>☐ Middle East<br>☐ Oceania (New Zealand, Papua New Guinea, Pacific Islands)<br>☐ Antarctica
Are you planning to travel overseas in the next 2 years?	◯ Yes<br>◯ No
	__________________________________
	__________________________________

Survey Completeness

20%

Part B: Past travel vaccines
International travel increases your chances of catching and spreading certain diseases. Some of these diseases, such as influenza, measles, hepatitis A, typhoid, Japanese encephalitis, rabies and yellow fever, can be prevented by vaccines. Vaccines given before an overseas trip are sometimes referred to as travel or pre-travel vaccines.

Have you ever seen a doctor, nurse or other health professional for advice about vaccines before an overseas trip?

◯ Yes
◯ No

Have you ever received vaccines before an overseas trip?

◯ Yes
◯ No

Which of the following vaccines have you received prior to an overseas trip?

	Yes	No	Not sure
Hepatitis A (Avaxim, Havrix, Twinrix, Vaqta or Vivaxim)	◯	◯	◯
Japanese encephalitis (Imojev or JEspect)	◯	◯	◯
Typhoid (Typhim, Vivotif or Vivaxim)	◯	◯	◯
Rabies (Merieux or Rabipur)	◯	◯	◯
Yellow fever (Stamaril)	◯	◯	◯

Where did you go to receive vaccines before your trip(s)? (Select all that apply)

☐ My regular general practice (GP) clinic
☐ A GP clinic different to my regular clinic
☐ A specialised travel clinic
☐ Other, please specify

Other location, please specify:

____

What prompted you to get vaccinated before your trip(s)? (please select all that apply)

☐ Recommended by family/friends
☐ Recommended by a health professional (e.g. GP, pharmacist)
☐ Required for entry into my destination / for visa purposes
☐ Required or recommended by my employer
☐ Destination seemed risky
☐ Other, please specify:

Other reason, please specify:

____

Why didn't you receive vaccines before your trip(s)? (select all that apply)	☐ I didn't think I was at risk<br>☐ I didn't think of it<br>☐ I didn't have enough time<br>☐ It was too inconvenient / I was too busy<br>☐ It was too expensive<br>☐ My doctor said I didn't need to<br>☐ My family/friends said I didn't need to<br>☐ I was travelling to a place I had been before<br>☐ I thought I was up to date with vaccines<br>☐ I hate needles<br>☐ Other, please specify
Other reason, please specify:	__________________________________
Have you had an influenza (flu) vaccine in the last 12 months?	◯ Yes<br>◯ No<br>◯ Not sure
Where did you go to receive the flu vaccine?	◯ GP clinic / community health centre<br>◯ Hospital / specialist rooms<br>◯ Pharmacy / chemist<br>◯ Workplace<br>◯ Other, please specify
Other location, please specify:	__________________________________
Survey Completeness<br><br>40%	

Part C: Hypothetical Scenarios

The following questions relate to two hypothetical scenarios. Please read the scenarios and answer the questions based on the information provided.

Scenario 1:
Imagine that you are planning a two-week overseas trip to a country where there is a risk of catching Disease X.

We know that:

Disease X is spread from person to person There is no risk of catching Disease X at home (the disease is not present in Australia The risk of catching Disease X during your two-week overseas trip is around 1 in 100 (1%) If you catch Disease X you have a 99 in 100 (99%) chance of having a mild illness from which you will fully recover and a 1 in 100 (1%) chance of developing severe illness Severe illness is associated with a 1 in 100 (1%) chance of death
A vaccine is available for disease X. The vaccine:

Is reasonably effective, reducing the risk of catching Disease X by about 50% Provides protection for 6 months Is safe, causing only mild side effects (such as redness and swelling around the injection site) in some people Costs $20

Based on this scenario:

Would you want to receive vaccination against Disease X before you travel?	◯ Yes<br>◯ No<br>◯ Not sure
Do you think most of your close family and friends would want you to get vaccinated against Disease X before you travel?	◯ Yes<br>◯ No

How easy would it be for you to pay for the vaccine against Disease X? When you think about the cost, please consider any payments to the clinic, the cost of the vaccine ($20), the cost of getting there, plus the cost of taking time away from work.

Would you say...	◯ Not at all easy<br>◯ A little easy<br>◯ Moderately easy<br>◯ Very easy

Survey Completeness

50%

Hypothetical Scenarios

Scenario 2:

Imagine that you are planning a two-week overseas trip to a country where there is a risk of catching Disease Y. We know that:

Disease Y is spread by mosquitos to humans There is no risk of catching Disease Y at home (the disease is not present in Australia) The risk of catching Disease Y during your two-week trip is around 1 in 100,000 (0.001%) If you catch Disease Y you have a 1 in 2 (50%) chance of having a mild illness from which you
will fully recover and a 1 in 2 (50%) chance of developing severe illness Severe illness is associated with a 1 in 2 (50%) risk of death A vaccine is available for Disease Y. The vaccine:

Is highly effective, reducing the risk of catching Disease Y by >99.9% Provides lifelong protection Is generally safe, causing no or mild side effects in most people, but on rare occasions (1 in 1,000,000 or 0.0001%) can lead to death Costs $200 Based on this scenario:

Would you want to receive vaccination against Disease Y before you travel?

- ◯ Yes
- ◯ No
- ◯ Not sure

Do you think most of your close family and friends would want you to get vaccinated against Disease Y before you travel?

- ◯ Yes
- ◯ No

How easy would it be to pay for the vaccine against Disease Y? When you think about the cost, please consider any payments to the clinic, the cost of the vaccine ($200), the cost of getting there, plus the cost of taking time away from work.

Would you say...

- ◯ Not at all easy
- ◯ A little easy
- ◯ Moderately easy
- ◯ Very easy

Survey Completeness

60%

Part D: Vaccine information and decision making

For each of the following statements concerning vaccines, please rate your level of agreement

	Strongly disagree	Disagree	Neutral	Agree	Strongly agree
Overall, I think vaccines are important for my health	○	○	○	○	○
Overall, I think vaccines are safe	○	○	○	○	○
Overall, I think vaccines are effective	○	○	○	○	○
I'd be more likely to get a vaccine if my family or friends have received it	○	○	○	○	○
I'd be more likely to get a vaccine if people in my community have received it	○	○	○	○	○
I prefer to make my own decisions about vaccines (independent of my doctor/health professional)	○	○	○	○	○
I prefer that my doctor/health professional makes decisions about vaccines for me	○	○	○	○	○
I prefer that my doctor/health professional and I share responsibility in decisions about vaccines	○	○	○	○	○

If you were looking for information or advice about travel vaccines, where would you go? (select all that apply)

- ☐ Internet
- ☐ General Practitioner (GP)
- ☐ Travel medicine specialist / Travel clinic
- ☐ Pharmacist / Chemist
- ☐ Travel agent
- ☐ Family / Friends
- ☐ Employer
- ☐ Community leader
- ☐ Religious leaders such as minister, pastor, priest or rabbi
- ☐ Other, please specify

Other source, please specify: __________________________________

How much do you trust vaccine information from the following sources?

	Not at all	A little	Moderately	Very much
Australian Government	◯	◯	◯	◯
Health professionals (e.g. doctors, nurses, pharmacists)	◯	◯	◯	◯
Community health centres / GP clinics	◯	◯	◯	◯
Specialised travel medicine clinics	◯	◯	◯	◯
Non-Government organisations (e.g. research institutes)	◯	◯	◯	◯
Social media	◯	◯	◯	◯
Travel agents	◯	◯	◯	◯
Family and friends	◯	◯	◯	◯
Community leaders	◯	◯	◯	◯
Religious leaders	◯	◯	◯	◯

Survey Completeness

75%

Part E: Vaccine decision aid

We are interested in developing a specific type of resource called a decision aid to help travellers make informed decisions about travel vaccines. Vaccine decision aids provide information on vaccines and the diseases they prevent, including risks and benefits of vaccination, how a person's age and personal health history may affect how their body responds to a disease or vaccine, and the pros and cons of different decisions.

An example of a decision aid developed to help people make decisions about COVID-19 vaccines is available here: https://www.ncirs.org.au/covid-19-decision-aid-for-adults

Have you used a decision aid before?
- ◯ Yes
- ◯ No

Did it help with your decision making?
- ◯ Yes
- ◯ No

Imagine that you need to make a decision about whether or not to get a certain vaccine. How important would the following information be in helping you make your decision?

	Not at all	A little	Moderately	Very much
Chance of getting the disease without vaccination (risk of disease)	◯	◯	◯	◯
Health risks of the disease (severity)	◯	◯	◯	◯
How the disease is spread	◯	◯	◯	◯
Availability of treatment for the disease	◯	◯	◯	◯
How the vaccine works	◯	◯	◯	◯
How long the vaccine provides protection for	◯	◯	◯	◯
Number of vaccine doses	◯	◯	◯	◯
Cost for all vaccine doses	◯	◯	◯	◯
How well the vaccine protects you against disease	◯	◯	◯	◯
Side effects of the vaccine	◯	◯	◯	◯
How the vaccine was tested	◯	◯	◯	◯

What additional information would you find most useful in a vaccine decision aid? __________________________________

Imagine that a decision aid for one or more travel vaccines was available. Please indicate your level of agreement with the following statements about using a travel vaccine decision aid

	Strongly disagree	Disagree	Neutral	Agree	Strongly agree
I would be interested in using a travel vaccine decision aid	○	○	○	○	○
I would prefer to use a travel vaccine decision aid on my own before seeing a health professional	○	○	○	○	○
I would prefer to use a travel vaccine decision aid with a family member or friend before seeing a health professional	○	○	○	○	○
I would prefer to use a travel vaccine decision aid with a trusted health professional (e.g. GP, nurse)	○	○	○	○	○

A decision aid can come in different formats. Please rate how likely you would be to use the following types of decision aids

	Unlikely	Neutral	Likely
Web-based (interactive site you can browse at your own pace)	◯	◯	◯
Video-based (video you can watch)	◯	◯	◯
Paper-based (printout you can write / draw on)	◯	◯	◯
PDF (digital version you can download and/or print)	◯	◯	◯

Is there anything else you would like to tell us?

____

Survey Completeness

90%

About you:

This final group of questions asks about you

What is your age (in years)?

____

What is your gender?

- ◯ Female
- ◯ Male
- ◯ Non-binary / gender diverse
- ◯ My gender identity isn't listed. I identify as...(please specify)
- ◯ Prefer not to say

Other gender, please specify:

____

What is your country of birth? (Please select from the list)

◯ Australia
◯ Afghanistan
◯ Albania
◯ Algeria
◯ American Samoa
◯ Andorra
◯ Angola
◯ Anguilla
◯ Antarctica
◯ Antigua and Barbuda
◯ Argentina
◯ Armenia
◯ Aruba
◯ Austria
◯ Azerbaijan
◯ Bahamas, The
◯ Bahrain
◯ Bangladesh
◯ Barbados
◯ Belarus
◯ Belgium
◯ Belize
◯ Benin
◯ Bermuda
◯ Bhutan
◯ Bolivia
◯ Bosnia and Herzegovina
◯ Botswana
◯ Bouvet Island
◯ Brazil
◯ British Indian Ocean Territory
◯ British Virgin Islands (BVI)
◯ Brunei
◯ Bulgaria
◯ Burkina Faso
◯ Burma
◯ Burundi
◯ Cambodia
◯ Cameroon
◯ Canada
◯ Cape Verde
◯ Cayman Islands
◯ Central African Republic
◯ Chad
◯ Chile
◯ China
◯ Christmas Island
◯ Cocos (Keeling) Islands
◯ Colombia
◯ Comoros
◯ Congo, DR
◯ Congo, Republic
◯ Cook Islands
◯ Costa Rica
◯ Cote d'Ivoire
◯ Croatia
◯ Cuba
◯ Curacao
◯ Cyprus
◯ Czech Republic
◯ Denmark
◯ Djibouti
◯ Dominica
◯ Dominican Republic
◯ Ecuador
◯ Egypt
◯ El Salvador
◯ Equatorial Guinea
◯ Eritrea

◯ Estonia
◯ eSwatini
◯ Ethiopia
◯ Falkland Islands (Islas Malvinas)
◯ Faroe Islands
◯ Fiji
◯ Finland
◯ France
◯ French Guiana
◯ French Polynesia
◯ French Southern and Antarctic Lands
◯ Gabon
◯ Gambia, The
◯ Gaza Strip
◯ Georgia
◯ Germany
◯ Ghana
◯ Gibraltar
◯ Greece
◯ Greenland
◯ Grenada
◯ Guadeloupe
◯ Guam
◯ Guatemala
◯ Guernsey
◯ Guinea
◯ Guinea-Bissau
◯ Guyana
◯ Haiti
◯ Heard Island and McDonald Islands
◯ Holy See (Vatican City)
◯ Honduras
◯ Hong Kong SAR
◯ Hungary
◯ Iceland
◯ India
◯ Indonesia
◯ Iran
◯ Iraq
◯ Ireland
◯ Isle of Man
◯ Israel
◯ Italy
◯ Jamaica
◯ Japan
◯ Jersey
◯ Jordan
◯ Kazakhstan
◯ Kenya
◯ Kiribati
◯ Korea, South
◯ Kosovo
◯ Kuwait
◯ Kyrgyzstan
◯ Laos
◯ Latvia
◯ Lebanon
◯ Lesotho
◯ Liberia
◯ Libya
◯ Liechtenstein
◯ Lithuania
◯ Luxembourg
◯ Macau SAR
◯ Madagascar
◯ Malawi
◯ Malaysia
◯ Maldives
◯ Mali
◯ Malta
◯ Marshall Islands

◯ Martinique
◯ Mauritania
◯ Mauritius
◯ Mayotte
◯ Mexico
◯ Micronesia, Federated States of
◯ Moldova
◯ Monaco
◯ Mongolia
◯ Montenegro
◯ Montserrat
◯ Morocco
◯ Mozambique
◯ Namibia
◯ Nauru
◯ Nepal
◯ Netherlands
◯ New Caledonia
◯ New Zealand
◯ Nicaragua
◯ Niger
◯ Nigeria
◯ Niue
◯ Norfolk Island
◯ North Macedonia
◯ Northern Mariana Islands
◯ Norway
◯ Oman
◯ Pakistan
◯ Palau
◯ Panama
◯ Papua New Guinea
◯ Paraguay
◯ Peru
◯ Philippines
◯ Pitcairn Islands
◯ Poland
◯ Portugal
◯ Puerto Rico
◯ Qatar
◯ Reunion
◯ Romania
◯ Russian Federation
◯ Rwanda
◯ Saint Barthelemy
◯ Saint Helena, Ascension, and Tristan da Cunha
◯ Saint Kitts and Nevis
◯ Saint Lucia
◯ Saint Martin
◯ Saint Pierre and Miquelon
◯ Saint Vincent and the Grenadines
◯ Samoa
◯ San Marino
◯ Sao Tome and Principe
◯ Saudi Arabia
◯ Senegal
◯ Serbia
◯ Seychelles
◯ Sierra Leone
◯ Singapore
◯ Sint Maarten
◯ Slovakia
◯ Slovenia
◯ Solomon Islands
◯ Somalia
◯ South Africa
◯ South Georgia and the Islands
◯ South Sudan
◯ Spain
◯ Sri Lanka
◯ Sudan

◯ Suriname
◯ Svalbard
◯ Sweden
◯ Switzerland
◯ Syria
◯ Taiwan
◯ Tajikistan
◯ Tanzania
◯ Thailand
◯ Timor-Leste
◯ Togo
◯ Tokelau
◯ Tonga
◯ Trinidad and Tobago
◯ Tunisia
◯ Turkey
◯ Turkmenistan
◯ Turks and Caicos Islands
◯ Tuvalu
◯ Uganda
◯ Ukraine
◯ United Arab Emirates
◯ United Kingdom
◯ United States
◯ Uruguay
◯ Uzbekistan
◯ Vanuatu
◯ Venezuela
◯ Vietnam
◯ Virgin Islands (US)
◯ Wallis and Futuna
◯ West Bank
◯ Western Sahara
◯ Yemen
◯ Zambia
◯ Zimbabwe

Other country not listed, please specify: ______________________________

Were both of your parents born in Australia?

◯ Yes
◯ No

In what country was your mother born? (Please select from the list)

◯ Australia
◯ Afghanistan
◯ Albania
◯ Algeria
◯ American Samoa
◯ Andorra
◯ Angola
◯ Anguilla
◯ Antarctica
◯ Antigua and Barbuda
◯ Argentina
◯ Armenia
◯ Aruba
◯ Austria
◯ Azerbaijan
◯ Bahamas, The
◯ Bahrain
◯ Bangladesh
◯ Barbados
◯ Belarus
◯ Belgium
◯ Belize
◯ Benin
◯ Bermuda
◯ Bhutan
◯ Bolivia
◯ Bosnia and Herzegovina
◯ Botswana
◯ Bouvet Island
◯ Brazil
◯ British Indian Ocean Territory
◯ British Virgin Islands (BVI)
◯ Brunei
◯ Bulgaria
◯ Burkina Faso
◯ Burma
◯ Burundi
◯ Cambodia
◯ Cameroon
◯ Canada
◯ Cape Verde
◯ Cayman Islands
◯ Central African Republic
◯ Chad
◯ Chile
◯ China
◯ Christmas Island
◯ Cocos (Keeling) Islands
◯ Colombia
◯ Comoros
◯ Congo, DR
◯ Congo, Republic
◯ Cook Islands
◯ Costa Rica
◯ Cote d'Ivoire
◯ Croatia
◯ Cuba
◯ Curacao
◯ Cyprus
◯ Czech Republic
◯ Denmark
◯ Djibouti
◯ Dominica
◯ Dominican Republic
◯ Ecuador
◯ Egypt
◯ El Salvador
◯ Equatorial Guinea
◯ Eritrea

◯ Estonia
◯ eSwatini
◯ Ethiopia
◯ Falkland Islands (Islas Malvinas)
◯ Faroe Islands
◯ Fiji
◯ Finland
◯ France
◯ French Guiana
◯ French Polynesia
◯ French Southern and Antarctic Lands
◯ Gabon
◯ Gambia, The
◯ Gaza Strip
◯ Georgia
◯ Germany
◯ Ghana
◯ Gibraltar
◯ Greece
◯ Greenland
◯ Grenada
◯ Guadeloupe
◯ Guam
◯ Guatemala
◯ Guernsey
◯ Guinea
◯ Guinea-Bissau
◯ Guyana
◯ Haiti
◯ Heard Island and McDonald Islands
◯ Holy See (Vatican City)
◯ Honduras
◯ Hong Kong SAR
◯ Hungary
◯ Iceland
◯ India
◯ Indonesia
◯ Iran
◯ Iraq
◯ Ireland
◯ Isle of Man
◯ Israel
◯ Italy
◯ Jamaica
◯ Japan
◯ Jersey
◯ Jordan
◯ Kazakhstan
◯ Kenya
◯ Kiribati
◯ Korea, South
◯ Kosovo
◯ Kuwait
◯ Kyrgyzstan
◯ Laos
◯ Latvia
◯ Lebanon
◯ Lesotho
◯ Liberia
◯ Libya
◯ Liechtenstein
◯ Lithuania
◯ Luxembourg
◯ Macau SAR
◯ Madagascar
◯ Malawi
◯ Malaysia
◯ Maldives
◯ Mali
◯ Malta
◯ Marshall Islands

◯ Martinique
◯ Mauritania
◯ Mauritius
◯ Mayotte
◯ Mexico
◯ Micronesia, Federated States of
◯ Moldova
◯ Monaco
◯ Mongolia
◯ Montenegro
◯ Montserrat
◯ Morocco
◯ Mozambique
◯ Namibia
◯ Nauru
◯ Nepal
◯ Netherlands
◯ New Caledonia
◯ New Zealand
◯ Nicaragua
◯ Niger
◯ Nigeria
◯ Niue
◯ Norfolk Island
◯ North Macedonia
◯ Northern Mariana Islands
◯ Norway
◯ Oman
◯ Pakistan
◯ Palau
◯ Panama
◯ Papua New Guinea
◯ Paraguay
◯ Peru
◯ Philippines
◯ Pitcairn Islands
◯ Poland
◯ Portugal
◯ Puerto Rico
◯ Qatar
◯ Reunion
◯ Romania
◯ Russian Federation
◯ Rwanda
◯ Saint Barthelemy
◯ Saint Helena, Ascension, and Tristan da Cunha
◯ Saint Kitts and Nevis
◯ Saint Lucia
◯ Saint Martin
◯ Saint Pierre and Miquelon
◯ Saint Vincent and the Grenadines
◯ Samoa
◯ San Marino
◯ Sao Tome and Principe
◯ Saudi Arabia
◯ Senegal
◯ Serbia
◯ Seychelles
◯ Sierra Leone
◯ Singapore
◯ Sint Maarten
◯ Slovakia
◯ Slovenia
◯ Solomon Islands
◯ Somalia
◯ South Africa
◯ South Georgia and the Islands
◯ South Sudan
◯ Spain
◯ Sri Lanka
◯ Sudan

- ◯ Suriname
- ◯ Svalbard
- ◯ Sweden
- ◯ Switzerland
- ◯ Syria
- ◯ Taiwan
- ◯ Tajikistan
- ◯ Tanzania
- ◯ Thailand
- ◯ Timor-Leste
- ◯ Togo
- ◯ Tokelau
- ◯ Tonga
- ◯ Trinidad and Tobago
- ◯ Tunisia
- ◯ Turkey
- ◯ Turkmenistan
- ◯ Turks and Caicos Islands
- ◯ Tuvalu
- ◯ Uganda
- ◯ Ukraine
- ◯ United Arab Emirates
- ◯ United Kingdom
- ◯ United States
- ◯ Uruguay
- ◯ Uzbekistan
- ◯ Vanuatu
- ◯ Venezuela
- ◯ Vietnam
- ◯ Virgin Islands (US)
- ◯ Wallis and Futuna
- ◯ West Bank
- ◯ Western Sahara
- ◯ Yemen
- ◯ Zambia
- ◯ Zimbabwe

Other country not listed, please specify: ______________________________

In what country was your father born? (Please select from the list)

- ◯ Australia
- ◯ Afghanistan
- ◯ Albania
- ◯ Algeria
- ◯ American Samoa
- ◯ Andorra
- ◯ Angola
- ◯ Anguilla
- ◯ Antarctica
- ◯ Antigua and Barbuda
- ◯ Argentina
- ◯ Armenia
- ◯ Aruba
- ◯ Austria
- ◯ Azerbaijan
- ◯ Bahamas, The
- ◯ Bahrain
- ◯ Bangladesh
- ◯ Barbados
- ◯ Belarus
- ◯ Belgium
- ◯ Belize
- ◯ Benin
- ◯ Bermuda
- ◯ Bhutan
- ◯ Bolivia
- ◯ Bosnia and Herzegovina
- ◯ Botswana
- ◯ Bouvet Island
- ◯ Brazil
- ◯ British Indian Ocean Territory
- ◯ British Virgin Islands (BVI)
- ◯ Brunei
- ◯ Bulgaria
- ◯ Burkina Faso
- ◯ Burma
- ◯ Burundi
- ◯ Cambodia
- ◯ Cameroon
- ◯ Canada
- ◯ Cape Verde
- ◯ Cayman Islands
- ◯ Central African Republic
- ◯ Chad
- ◯ Chile
- ◯ China
- ◯ Christmas Island
- ◯ Cocos (Keeling) Islands
- ◯ Colombia
- ◯ Comoros
- ◯ Congo, DR
- ◯ Congo, Republic
- ◯ Cook Islands
- ◯ Costa Rica
- ◯ Cote d'Ivoire
- ◯ Croatia
- ◯ Cuba
- ◯ Curacao
- ◯ Cyprus
- ◯ Czech Republic
- ◯ Denmark
- ◯ Djibouti
- ◯ Dominica
- ◯ Dominican Republic
- ◯ Ecuador
- ◯ Egypt
- ◯ El Salvador
- ◯ Equatorial Guinea
- ◯ Eritrea

◯ Estonia
◯ eSwatini
◯ Ethiopia
◯ Falkland Islands (Islas Malvinas)
◯ Faroe Islands
◯ Fiji
◯ Finland
◯ France
◯ French Guiana
◯ French Polynesia
◯ French Southern and Antarctic Lands
◯ Gabon
◯ Gambia, The
◯ Gaza Strip
◯ Georgia
◯ Germany
◯ Ghana
◯ Gibraltar
◯ Greece
◯ Greenland
◯ Grenada
◯ Guadeloupe
◯ Guam
◯ Guatemala
◯ Guernsey
◯ Guinea
◯ Guinea-Bissau
◯ Guyana
◯ Haiti
◯ Heard Island and McDonald Islands
◯ Holy See (Vatican City)
◯ Honduras
◯ Hong Kong SAR
◯ Hungary
◯ Iceland
◯ India
◯ Indonesia
◯ Iran
◯ Iraq
◯ Ireland
◯ Isle of Man
◯ Israel
◯ Italy
◯ Jamaica
◯ Japan
◯ Jersey
◯ Jordan
◯ Kazakhstan
◯ Kenya
◯ Kiribati
◯ Korea, South
◯ Kosovo
◯ Kuwait
◯ Kyrgyzstan
◯ Laos
◯ Latvia
◯ Lebanon
◯ Lesotho
◯ Liberia
◯ Libya
◯ Liechtenstein
◯ Lithuania
◯ Luxembourg
◯ Macau SAR
◯ Madagascar
◯ Malawi
◯ Malaysia
◯ Maldives
◯ Mali
◯ Malta
◯ Marshall Islands

◯ Martinique
◯ Mauritania
◯ Mauritius
◯ Mayotte
◯ Mexico
◯ Micronesia, Federated States of
◯ Moldova
◯ Monaco
◯ Mongolia
◯ Montenegro
◯ Montserrat
◯ Morocco
◯ Mozambique
◯ Namibia
◯ Nauru
◯ Nepal
◯ Netherlands
◯ New Caledonia
◯ New Zealand
◯ Nicaragua
◯ Niger
◯ Nigeria
◯ Niue
◯ Norfolk Island
◯ North Macedonia
◯ Northern Mariana Islands
◯ Norway
◯ Oman
◯ Pakistan
◯ Palau
◯ Panama
◯ Papua New Guinea
◯ Paraguay
◯ Peru
◯ Philippines
◯ Pitcairn Islands
◯ Poland
◯ Portugal
◯ Puerto Rico
◯ Qatar
◯ Reunion
◯ Romania
◯ Russian Federation
◯ Rwanda
◯ Saint Barthelemy
◯ Saint Helena, Ascension, and Tristan da Cunha
◯ Saint Kitts and Nevis
◯ Saint Lucia
◯ Saint Martin
◯ Saint Pierre and Miquelon
◯ Saint Vincent and the Grenadines
◯ Samoa
◯ San Marino
◯ Sao Tome and Principe
◯ Saudi Arabia
◯ Senegal
◯ Serbia
◯ Seychelles
◯ Sierra Leone
◯ Singapore
◯ Sint Maarten
◯ Slovakia
◯ Slovenia
◯ Solomon Islands
◯ Somalia
◯ South Africa
◯ South Georgia and the Islands
◯ South Sudan
◯ Spain
◯ Sri Lanka
◯ Sudan

◯ Suriname
◯ Svalbard
◯ Sweden
◯ Switzerland
◯ Syria
◯ Taiwan
◯ Tajikistan
◯ Tanzania
◯ Thailand
◯ Timor-Leste
◯ Togo
◯ Tokelau
◯ Tonga
◯ Trinidad and Tobago
◯ Tunisia
◯ Turkey
◯ Turkmenistan
◯ Turks and Caicos Islands
◯ Tuvalu
◯ Uganda
◯ Ukraine
◯ United Arab Emirates
◯ United Kingdom
◯ United States
◯ Uruguay
◯ Uzbekistan
◯ Vanuatu
◯ Venezuela
◯ Vietnam
◯ Virgin Islands (US)
◯ Wallis and Futuna
◯ West Bank
◯ Western Sahara
◯ Yemen
◯ Zambia
◯ Zimbabwe

Other country not listed, please specify: ____________________

Do you speak a language other than English at home?

◯ No - English only
◯ Yes - Other, please specify

Other language, please specify: ____________________

Are you of Aboriginal or Torres Strait Islander origin, or both?

◯ No
◯ Yes - Aboriginal
◯ Yes - Torres Strait Islander
◯ Yes - Both Aboriginal and Torres Strait Islander

What is the highest educational qualification you have completed?

◯ Primary school
◯ Secondary / high school
◯ Trade or TAFE qualification
◯ Undergraduate (Bachelors) degree
◯ Postgraduate degree

Which State / Territory do you currently live in?	◯ ACT<br>◯ New South Wales<br>◯ Northern Territory<br>◯ Queensland<br>◯ South Australia<br>◯ Tasmania<br>◯ Victoria<br>◯ Western Australia
What is your employment status presently?	◯ Employed full-time<br>◯ Employed part-time<br>◯ Employed casually / hourly<br>◯ In a family business without pay<br>◯ Home maker<br>◯ Student<br>◯ Unpaid voluntary work<br>◯ Unemployed<br>◯ Retired
Do you have private health insurance?	◯ Yes<br>◯ No
In general, how would you rate your health today?	◯ Very good<br>◯ Good<br>◯ Moderate<br>◯ Poor<br>◯ Very poor
Survey Completeness<br><br>100%	
